# The characteristics of mixing patterns of sexual dyads and factors correlated with condomless anal intercourse among men who have sex with men in Guangzhou, China

**DOI:** 10.1186/s12889-019-7082-9

**Published:** 2019-06-10

**Authors:** Juan Yang, Huifang Xu, Shuo Li, Weibin Cheng, Yuzhou Gu, Peng Xu, Qiuyan Yu, Fan Lv

**Affiliations:** 1 The National Center for AIDS/STD Control and Prevention, Chinese Center for Disease Control and Prevention, 155th Changbai Road, Changping District, Beijing, China; 2 Department of HIV/AIDS Control and Prevention, Center for Disease Control and Prevention, Guangzhou, China; 3 United Nations Entity for Gender Equality and the Empowerment of Woman, China Office, Beijing, China

**Keywords:** HIV MSM egocentric network CAI

## Abstract

**Background:**

The HIV prevalence among men who have sex with men (MSM) in China has increased yearly. This study aimed to explore the association between the characteristics of social communication and condomless anal intercourse (CAI) among MSM and the implications for prevention and control of HIV among MSM in China using an egocentric network framework.

**Methods:**

The data were collected in Guangzhou between November 2016 and May 2017 through standardized face-to-face interviews. The participants were recruited among MSM who received voluntary counselling and testing services (VCT) provided by nongovernmental organizations (NGOs) and the local Centers for Disease Control and Prevention (CDC). We used the framework of the egocentric network analysis, the odd ratios of CAI were analyzed using generalized estimating equations (GEE).

**Results:**

In total, 1073 MSM who nominated 2667 sexual partners were sampled. MSM who were approximately 30 years old and chose sexual partners of different age category were more likely to engage in CAI. Participants with high level education who were in partnerships with individuals with lower education levels had a higher risk of CAI. Participants who reported having a strong relationship with their sexual partners(AOR = 1.31) were associated with a higher probability of experiencing CAI during sex; while having sexual partners who were unmarried (OR = 0.56), and participants who reported meeting sexual partners online (AOR = 0.74) or, having sex with an occasional partner (AOR = 0.44)were less likely to engage in CAI.

**Conclusion:**

Our study indicates that the strength of sexual dyadic relational ties and different social communication mixing patterns across ages, educational categories, and marital status were associated with CAI.

**Electronic supplementary material:**

The online version of this article (10.1186/s12889-019-7082-9) contains supplementary material, which is available to authorized users.

## Background

Men who have sex with men (MSM) are heavily affected by the HIV/AIDS epidemic in China. Between 2012 and 2017, the annual number of new diagnoses among MSM increased from 15,768 to 34,358 [1, 2]. According to the annual sentinel surveillance survey conducted by the National Center for AIDS/STD Control and Prevention in China, the HIV prevalence over the years among MSM increased from 0.9% in 2003 to 7.75% in 2014 [3, 4], indicating an increasing trend of HIV prevalence among MSM community members.

Recently, the Chinese government implemented aggressive HIV prevention and intervention programs targeting MSM regarding HIV transmission [5, 6]. Although the implementation of HIV prevention interventions in China has achieved substantial results in recent years, and the proportion of condom use among sexual partners of MSM has increased, adherence to condom use is difficult to achieve [7]. Therefore, the target of containing the spread of the HIV epidemic among MSM has not met expectation. Several factors are responsible for the slow rate of large-scale changes in behavior, such as the early decay rate of newly learnt behaviors which is dependent on the mode of learning among others. However, the specific social communication characteristics of sexual networks among MSM who usually engage in condomless anal intercourse (CAI) with their sexual partners may play an important role. According to a meta-analysis conducted by Hess KL, CAI with an HIV-unknown partner has become an increasing trend among MSM [8], and the increasing trend of HIV-related risk behaviours may help explain the increase in the HIV epidemic.

Numerous studies have explored the characteristics of CAI and its association with MSM to provide practical guidance for targeted policy solutions. Most studies have focused on the demographic characteristics and their relationship to CAI. For example, a young age, lower education level, and being divorced have been shown to be risk factors of CAI [9]. But it is not known why these factors are more likely to be associated with CAI, it may be related to the social communication characteristics among MSM, that is, who, where, and how MSM meet sexual partners during social communication. Ying Wang et al. found that psychosocial problems were associated with CAI among MSM [10]. According to Yong Cai, sexual partner type is highly associated with CAI, and the prevalence of having CAI with regular partners is high among MSM [11]. The CAI risk factors are multidimensional; however, these studies did not present the social communication characteristics and their relationship to CAI among MSM. Notably, social communication studies exploring CAI among MSM are needed to identify the enablers of HIV transmission among MSM. Fortunately, social and sexual contact networks among MSM play an important role in understanding HIV transmission [12]. The social network analysis method produces a more comprehensive understanding of the MSM population and provides a good method for interpreting behavior. In social networks, each individual is a node that can connect with one or more nodes directly or indirectly, and the high-risk behaviors of these nodes makes them more susceptible to HIV infection, resulting in extensive transmission of HIV within the network.

The social network concept first appeared in HIV-related research in 1985 [13]. The rising HIV epidemic among MSM was used by multiple scholars to understand and construct HIV prevention and intervention schemes. For example, one study reported that changing risk behaviors via an intervention program conducted by a leader of a social network among MSM was effective [14]. While concerns about the relationship between risky behaviours and social network characteristics are increasing, as this relationship is closely associated with the HIV epidemic in many countries [15, 16], very few articles have analyzed the factors correlated with CAI from the perspective of an egocentric network, which mainly focuses on social relationships and the mixing patterns associated with social communication in a large sample in China.

This study adopted an egocentric network framework to explore the characteristics of mixing patterns of sexual dyads and factors correlated with CAI among MSM and the implications for the prevention and control of HIV in China.

## Methods

### Participants and recruitment

We conducted a cross-sectional survey through standardized face-to-face interviews in Guangzhou, which is a city in South-eastern China with over 12 million residents. An estimated 44,593 sexually active MSM live in the city in 2008 [17]. The HIV prevalence among MSM in Guangzhou significantly increased from 5.0% (19/379) in 2008 to 11.4% (72/633) in 2013 [17]. The participants were limited to MSM who received voluntary counselling and testing services (VCT) provided by nongovernmental organizations (NGOs) and the local Centesr for Disease Control and Prevention (CDC) between November 2016 and May 2017. The inclusion criteria were as follows: (1) men who self-reported having had anal sex with at least one man in the prior 12 months; (2) age of 18 years or above; (3) ability to provide written and verbal consent in Mandarin Chinese; (4) no previous HIV positive diagnosis; and (5) voluntarily received VCT for an HIV test. The exclusion criteria were as follows: (1) female; (2) age less than 18 years; (3) could not understand or reply to questions; (4) diagnosed with HIV infection; and (5) had not lived in Guangzhou for more than 3 months.

### Interview procedures

Our study used the traditional paper-and-pencil interview methodology. The participants were asked to complete a questionnaire in private with assistance from trained interviewers from the district-level CDC and local NGOs. The participants completed the survey within an average of 25 min. A small gift was given to the respondents for their participation.

### Measures

#### Ego-network data collection

Regardless of whether they engaged in receptive or insertive anal sex, the respondents were asked to name (by simply listing a nickname) one or more male sexual partners with whom they have had sex with in the prior 12 months and report their own and their partners’ demographic backgrounds (i.e., age, education, income, marital status, occupation, and frequency of HIV testing). The participants were also asked about the social ties of their sexual dyads in terms of three constructs (i.e., the duration and frequency of the interaction and intimacy) and other features of social communication (i.e., the frequency of having sex with sexual partners, the frequency of condom usage during sex, method of seeking partners, etc.) (Additional file 1).

#### Social tie strength (STS)

The participants were asked to answer 3 specific questions about their relationships with their sexual partners. The questions were related to (1) the duration of the social interaction, (2) the frequency of the social interaction with the partner, and (3) the affective interaction with the partner (intimacy). For each question, the participants could choose from the following options: 1 (weak) to 5 (strong). The items were scored on a Likert scale from 1 through 5. We classified the social ties into two groups, i.e., strong and weak, based on Granovetter’s study [18]. In this paper, the group that scored 9 or above was defined as having strong ties, while the group that scored less than 9 was defined as having weak ties (Additional file 1).

#### Condomless anal intercourse

Participants stated whether they had had condomless insertive anal intercourse without using HIV-PrEP (Pre-Exposure Prophylaxis) with sexual partners in the past 12 months. There were four options: (1) never used a condom; (2) seldom used condoms; (3) intermittent used condoms; (4) always used condoms. In this study, we considered the participants who chose option 1,2 and 3 as having had CAI (Additional file 1).

#### Occasional sexual partners

Occasional sexual partners were defined as individuals who had had only one sexual intercourse experience with the nominated partners. The question was related to how many times they had had sex with this partner in last 12 months, for this question, the participants could choose (1) just once; (2) less than once each month; (3) at least once each month; (4) at least once each week; or (5) almost every day (Additional file 1).

#### Place of first date

Place of first date was classified as a dichotomous variable: private or public place. A private place was defined as the first date occurring in a private residence or hotel where no one else was present; a public place was defined as a location where other people were present. For the question asking where did MSM meet each other when they were first dating, the answer options were (1) wine bar or KTV; (2) chess room or tea room; (3) public bathhouse or clubhouse; (4) public park; (5) private residence or hotel; and (6) shopping mall or a public entertainment venue (Additional file 1).

#### Method of meeting sexual partners

We defined the method of meeting sexual partners as a dichotomous variable: Internet or non-Internet. Participants who indicated that they met sexual partners via dating software or websites were classified in the Internet group; conversely, participants who were introduced to sexual partner through other non-internet channels were classified in the non-Internet group. The question was “Do you meet with your sexual partners via dating software or websites? with answer options of 1=yes, 0=no” (Additional file 1).

### Statistical analyses

#### Social network analysis

Social network analyses can include whole and ego-network analyses based on different objectives. In this study, we used the framework of the egocentric network analysis, which mainly focuses on STS and mixing patterns among the study respondents and their sexual partners, to explore the characteristics and correlation factors of CAI.

#### Mixing matrices

A mixing matrix was compiled by creating a table of the types of partnerships within two groups: respondents who engaged in CAI and non-CAI with partners. Each sexual dyad was assigned into mixing matrices [19] by cross-classification according to demographic characteristics, including age, education level, income and marital status, of the respondents (row) and their indicated partners (columns). A Q value computed from eigenvalues was used to test the concordance patterns. Although this measure is a continuous value, it is often applied as a categorical assessment, for which any positive value indicates concordance mixing and all negative values indicate disconcordance patterns [20].

#### Chi square test

The variables obtained from the univariate analysis were identified as candidates by a Chi square test (entry *P <* 0.1; removal *P* ≥ 0.1).

#### Regression analysis

The GENMOD procedure fits generalized linear models; the class of generalized linear models is an extension of traditional linear models that allows the mean of a population to depend on a linear predictor through a nonlinear link function and allows the response probability distribution to be any member of an exponential family of distributions. The GENMOD procedure can fit models to correlated responses by the GEE method. The sexual dyad was the analysis unit in this study. Because 72.5% of the 1073 respondents nominated at least two sexual partners, each participant may be a part of multiple dyads; thus, there may be correlations between members from different dyads. We used robust generalized estimating equations (GEE) [21] to analyze the relative CAI risk factors in this paper using correlated dyadic data clustered among the respondents. The REPEATED statement invokes the GEE method. In this model, CAI is the dependent variable, and we used the ID number of participants as the repeated subject. All analyses were conducted using SAS V.9.4.

## Results

In total, 1073 respondents who nominated 2667 sexual partners were interviewed face-to-face. In total, 41.58% of the 2667 sexual dyads reported that they have engaged in CAI in the previous twelve months (Additional file 2).

### Demographic characteristics of the study respondents and their sexual partners

Most participants and their partners, i.e., 68.50 and 74.58%, respectively, were under the age of 30 years. In total, 62.35% of the participants and 57.74% of the sexual partners had attended a college or university, and 84.16% of the participants and 78.52% of the sexual partners had not been married, while 57.41% of the participants and 55.0% of the sexual partners earned middle level incomes (Table 1).

**Table 1:** Background characteristics of the respondents and their sexual partners

Characteristics	Socio-demographics of the respondents (*N* = 1073)		Socio-demographics of the sexual partners (*N* = 2667)	
	*n*	%	*n*	%
Age group				
≤ 17	0	0	25	0.94
18–25	430	40.07	1053	39.48
26–30	305	28.43	911	34.16
31–40	243	22.65	487	18.26
41–50	79	7.36	146	5.47
> 51	16	1.49	45	1.69
Education background				
Primary school	1	0.09	13	0.49
Middle school	100	9.32	206	7.72
High school	202	18.83	539	20.21
Undergraduate school	669	62.35	1540	57.74
Master’s or above	101	9.41	198	7.42
Unknown	0	0	171	6.41
Marital status				
Married	142	13.23	467	17.51
Unmarried	903	84.16	2094	78.52
Divorced	26	2.42	71	2.66
Other	2	0.19	35	1.31
Income (12 months)				
< 20,000	287	26.75	400	15.00
20,000-	322	30.01	689	25.83
60,001-	294	27.40	778	29.17
120,001-	136	12.67	346	12.97
> 240,000	34	3.17	119	4.46
Unknown	0	0	335	12.56

### Characteristics of the social network of the study respondents and their sexual partners

#### Social tie strength

The highest score, i.e., 15, accounted for 0.26% of the 2667 sexual dyads, while 289 (10.84%) dyads obtained the lowest score, i.e., 3. In general, the group with strong ties scored 9 and above and included 1117 (41.88%) sexual dyads, while the group with weak ties scored under 9 and included 1550 (58.12%) sexual dyads.

#### Mixing patterns among the study respondents and their sexual partners

Table 2 (located at the end of this text) shows the percentage distribution by age, education, income, and marital status among respondents who engaged in CAI and non-CAI with partners based on the demographic groupings of the respondents. In most categories of most variables, the study participants reported having the largest number of sexual partners in the same category as themselves (for example, the proportion of 18–25 years old CAI partners of the all CAI partners of 18–25 years old participants was 55.49%). The exact proportion of sexual partners who were concordant with the participants varied across the categories of age, educational level, income and marital status. There were statistically significant differences in the distribution of age, educational level, income and marital status according to whether CAI occurred (*P <* 0.01). In general, the distribution of partnership concordance in education (Q = 0.2) was greater than that in the other variables, while the proportion of age (Q = 0.11) showed a lower concordance.

**Table 2:** Patterns among the participants and their sexual partners

Participants characteristic	Total, %		Partners, %											
	Non-CAI	CAI	Non-CAI	CAI	Non-CAI	CAI	Non-CAI	CAI	Non-CAI	CAI	Non-CAI	CAI	Non-CAI	CAI
Age, y			under 17		18–25		26–30		31–40		41–50		≥51	
18–25	572 (54.22)	483 (45.78)	3 (0.52)	14 (2.9)	328(57.34)	268(55.49)	155 (27.1)	121(25.05)	57 (9.97)	56 (11.59)	23 (4.02)	15 (3.11)	6 (1.05)	9 (1.86)
26–30	452 (61.33)	285 (38.67)	2 (0.44)	3 (1.05)	138(30.53)	103(36.14)	206 (45.58)	106(37.19)	88 (19.47)	55 (19.3)	10 (2.21)	14 (4.91)	8 (1.77)	4 (1.4)
31–40	377 (61.90)	232 (38.10)	1 (0.27)	1 (0.43)	111(29.44)	48 (20.69)	145 (38.46)	105(45.26)	98 (25.99)	53 (22.84)	21 (5.57)	20 (8.62)	1 (0.27)	5 (2.16)
41–50	137 (60.89)	88 (39.11)	0 (0.0)	1 (1.14)	29 (21.17)	21 (23.86)	40 (29.2)	26 (29.55)	42 (30.66)	22 (25.0)	20 (14.6)	14 (15.91)	6 (4.38)	4 (4.55)
≥51	20 (48.78)	21 (51.22)	0 (0.0)	0 (0.0)	2 (10.0)	5 (23.81)	5 (25.0)	2 (9.52)	5 (25.0)	11 (52.38)	7 (35.0)	2 (9.52)	1 (5.0)	1 (4.76)
Education			primary school		middle school		high school		undergraduate school		Master’s and above		unknown	
primary school	1 (100.00)	0 (0)	0 (0)	0 (0)	0 (0)	0 (0)	0 (0)	0 (0)	1 (100)	0 (0)	0 (0)	0 (0)	0 (0)	0 (0)
middle school	131 (49.25)	135 (50.75)	1 (0.76)	4 (2.96)	43 (32.82)	49 (36.3)	36 (27.48)	33 (24.44)	32 (24.43)	27 (20.0)	2 (1.53)	4 (2.96)	17 (12.98)	18 (13.33)
high school	285 (57.11)	214 (42.89)	1 (0.35)	2 (0.93)	27 (9.47)	23 (10.75)	106(37.19)	90 (42.06)	118 (41.4)	74 (34.58)	16 (5.61)	10 (4.67)	17 (5.96)	15 (7.01)
undergraduate school	995 (60.34)	654 (39.66)	3 (0.3)	2 (0.31)	26 (2.61)	31 (4.74)	141(14.17)	108 (16.51)	689 (69.25)	441 (67.43)	76 (7.64)	35 (5.35)	60 (6.03)	37 (5.66)
Master’s and above	146 (57.94)	106 (42.06)	0 (0)	0 (0)	5 (3.42)	2 (1.89)	10 (6.85)	15 (14.15)	86 (58.9)	72 (67.92)	40 (27.4)	15 (14.15)	5 (3.42)	2 (1.89)
Income, yuan/year			≤20,000		20,001-		60,001-		120,001-		≥240,001		unknown	
≤20,000	377 (53.63)	326 (46.37)	124 (32.89)	111(34.05)	81 (21.49)	89 (27.3)	79 (20.95)	49 (15.03)	26 (6.9)	26 (7.98)	11 (2.92)	5 (1.53)	56 (14.85)	46 (14.11)
20,001-	481 (57.74)	352 (42.26)	55 (11.43)	45 (12.78)	190 (39.5)	139(39.49)	128 (26.61)	90 (25.57)	38 (7.9)	27 (7.67)	8 (1.66)	7 (1.99)	62 (12.89)	44 (12.5)
60,001-	426 (60.51)	278 (39.49)	18 (4.23)	22 (7.91)	93 (21.83)	59 (21.22)	179 (42.02)	118 (42.45)	64 (15.02)	37 (13.31)	17 (3.99)	8 (2.88)	55 (12.91)	34 (12.23)
120,001-	223 (63.90)	126 (36.10)	11 (4.93)	7 (5.56)	21 (9.42)	14 (11.11)	76 (34.08)	36 (28.57)	67 (30.04)	45 (35.71)	27 (12.11)	17 (13.49)	21 (9.42)	7 (5.56)
≥240,001	51 (65.38)	27 (34.62)	5 (9.8)	2 (7.41)	3 (5.88)	0 (0)	15 (29.41)	8 (29.63)	8 (15.69)	8 (29.63)	13 (25.49)	6 (22.22)	7 (13.73)	3 (11.11)
Marital status			Married		Unmarried		Divorced		Separated		other			
Married	204 (56.51)	157 (43.49)	83 (40.69)	82 (52.23)	112 (54.9)	62 (39.49)	4 (1.96)	9 (5.73)	3 (1.47)	0 (0)	2 (0.98)	4 (2.55)		
Unmarried	1299 (58.49)	922 (41.51)	167(12.86)	112(12.15)	1092 (84.06)	768 (83.3)	29 (2.23)	27 (2.93)	2 (0.15)	2 (0.22)	9 (0.69)	13 (1.41)		
Divorced	51 (62.96)	30 (37.04)	17 (33.33)	5 (16.67)	33 (64.71)	24 (80.0)	1 (1.96)	1 (3.33)	0 (0)	0 (0)	0 (0)	0 (0)		
Other	4 (100.00)	0 (0)	1 (25.0)	0 (0)	3 (75.0)	0 (0)	0 (0)	0 (0)	0 (0)	0 (0)	0 (0)	0 (0)		

Note. Denominators: the number of the all CAI/non-CAI partners of participants respect to each demographic category

Numerators: the number of each demographic category among the denominators

The greatest proportion of concordant sexual partnerships (include CAI and non-CAI) with respect to age was reported by 18–25 years old (56.49%), followed by 26–30 years old (42.33%). The lowest concordance in the age category was reported by those who were 51 years or older (4.88%). MSM who had an undergraduate education reported the greatest percentage of sexual partnership concordance (68.53%), followed by those with high school education (39.28%). The greatest proportion of concordance across income and marital status were 42.19 and 83.75% among MSM with income of 60,001–120,000 and unmarried respectively.

The proportion of CAI varied according to respondent characteristics, participants of 51 years or older (51.22%), with middle school education(50.75%), lower income (≤20,000) (46.37%) and, who were married(43.49%) were more likely to engage in CAI.

#### Mixing patterns and CAI risk (risk factor analysis using a regression model)

The mixing patterns across MSM from different age groups, educational levels and marital statuses were significantly associated with the practise of CAI. We analyzed the age, education and marital status of participants and respondents separately in the regression models.

18 to 25 years old MSM were more likely to engage CAI with younger than 18 years old sexual partners (OR = 5.71, *P* < 0.01), respondents who were 26 to 30 years old and partnered with 18- to 25-year-olds and 41- to 50-year-olds had elevated risks of CAI (OR = 1.45, *P* < 0.05, and OR = 2.72, *P* < 0.05). MSM aged 31 to 40 years who reported partnerships with 51-year-olds and above were at an increased risk of CAI (OR = 9.07, *P* < 0.05).

Mixing across educational categories among the highly educated groups was associated with an increased CAI risk. Participants who reported partnerships with people who were less educated than themselves had an increased CAI risk (OR = 1.86, *P* < 0.05, OR = 4.0, *P* < 0.01, OR = 2.23, *P* < 0.05).

Among the MSM who were married to women, having homosexual partners who were not married to women was a protective factor against CAI (OR = 0.56, *P* < 0.01) (Table 3).

**Table 3:** ORs of CAI among participants with concordant/discordant partners within age, education and marital status

Respondents’ characteristics	Odds Ratio of CAI^#^ 95% CI					
Age, yn	≤17	18-	26-	31-	41-	≥ 51
18-	5.71**(1.62–20.08)	1.00^‡^	0.96(0.72–1.27)	1.20(0.80–1.80)	0.80(0.41–1.56)	1.84(0.65–5.22)
26-	2.92 (0.48–17.71)	1.45*(1.03–2.05)	1.00^‡^	1.21 (0.81–1.83)	2.72*(1.17–6.33)	0.97(0.29–3.30)
31-	1.81 (0.11–29.59)	0.77 (0.48–1.24)	1.31 (0.87–1.99)	1.00^‡^	1.73 (0.86–3.47)	9.07*(1.07–79.67)
41-	–	1.03 (0.43–2.50)	0.93(0.40–2.16)	0.75(0.32–1.76)	1.00^‡^	0.95(0.23–4.01)
≥51	–	2.50 (1.0–62.60)	0.40 (0.02–10.02)	2.20 (0.11–42.73)	0.29 (0.01–6.91)	1.00^‡^
Education background	Primary school	Middle school	High school	Undergraduate	Master’s and above	Unknown
Middle school	3.51(0.38–32.62)	1.00^‡^	0.80(0.43–1.50)	0.72(0.37–1.38)	1.76(0.31–10.06)	0.93(0.43–2.03)
High school	2.36(0.22–51.14)	1.00(0.53–1.87)	1.00^‡^	0.74(0.49–1.11)	0.74(0.31–1.68)	1.04(0.49–2.20)
Undergraduate	1.04(0.14–6.31)	1.86*(1.09–3.20)	1.20(0.91–1.58)	1.00^‡^	0.72(0.47–1.08)	0.96(0.62–1.47)
Master’s and above	–	1.07(0.14–5.56)	4.00**(1.50–11.16)	2.23*(1.16–4.47)	1.00^‡^	1.07(0.14–5.56)
Marital status	Married	Unmarried	Divorced			
Married	1.00^‡^	0.56**(0.13–0.98)	2.28(1.06–3.49)			
Unmarried	0.95(0.74–1.23)	1.00^‡^	1.32(0.77–2.26)			
Divorced	0.31(0.01–8.87)	0.73(0.03–18.99)	1.00^‡^			

Note. *P < 0.05 and **P < 0.01. # odds ratio of CAI refers to compare the odds of the discordant mixing groups to the odds of the concordant mixing groups

‡ reference of each analysis model. —— have no enough data in this group

#### Characteristics of social relationships and CAI risk

We used multiple variable analyses, except for the variable lists in Table 4, We adjusted for age, educational background and marital status of the participants and their partners. The regression analysis showed that the dyadic relationship characteristics (STS, partner type, and method of meeting sexual partners) were independently associated with CAI. Partners who had a strong relationship with the study participants (AOR = 1.31) were associated with a higher chance of having CAI, while meeting sexual partners via the Internet (AOR = 0.74) and having had sex with an occasional partner (AOR = 0.44) were considered protective factors (Table 4).

**Table 4:** Association between the characteristics of the sexual dyads and CAI (dyads = 2667)

Relationship characteristics	Estimate	Z	AOR^a^	95% CI		*P*-value
STS						
Weak			1.00			
Strong	0.2706	2.65	1.31	1.07	1.60	0.008
Partner type						
Regular partner			1.00			
Occasional partner	−0.8159	−7.73	0.44	0.36	0.54	< 0.0001
Method of meeting sexual partners						
Non-Internet			1.00			
Internet	−0.2981	−2.66	0.74	0.60	0.92	0.008

^a^We have adjusted for age, educational background and marital status of the participants and their partners

## Discussion

We examined the relationship and social communication characteristics for similarities and differences among the participants and their sexual partners in detail in this study.

We observed that different partner typologies were differentially related to the occurrence of CAI. Previous studies have corroborated the conclusion that young MSM have a higher risk of having CAI with sexual partners during anal intercourse [9] [22]. However, our study identified other influencing factors. Based on our risk factor analysis, frequently, MSM who were approximately 30 years old and chose younger or older (41 years or older) partners were more likely to engage in CAI than those who chose sexual partners of their same age category. We also found no significant differences in the prevalence of CAI among MSM who were 41 years of age or older with sexual partners from any age category. This condom using imbalance need further exploring whether it is related to more bargaining power during sex for seniority in age. Younger MSM who have sex with older partners may have an increased risk of HIV infection due to the increased HIV prevalence among older sexual partners [23]. Thus, we should not only strengthen preventative interventions and education campaigns targeting young MSM but also implement targeted interventions and campaigns to instil a sense of responsibility in older MSM and to encourage these MSM to use protective measures during sex.

Measurable differences were also reported in the risk of CAI across the education level and marital status categories. A high rate of CAI was associated with particular mixing patterns. Participants with college-level or above education who were in partnerships with individuals with lower education levels had a higher risk of CAI. To further investigate this association, we found that having sexual partners with higher education levels (compared to the respondents) presented a protective factor against practicing CAI. Zhongrong Y et al. reported a high CAI prevalence among MSM who were college students in China [22], However, MSM with higher education levels are well-known to have a higher HIV-related knowledge score [24]. This imbalance may stem from that MSM may avoid using condom in order to cope with psychosocial vulnerabilities and create intimacy with their partners [25], when they have sex with assertive partners who may lack of knowledge about HIV and resistant to use condom. To address this imbalance in awareness between partners of different education levels, the coverage of HIV-related knowledge awareness campaigns needs to be expanded to primary and middle schools.

We also found that within the category of married respondents, significant differences existed in condom usage among sexual partners with different marital statuses. Married MSM were more likely to choose other married MSM as their sexual partners, and this combination was associated with CAI. Few relevant results have been reported in previous studies, as other researchers have focused more on individual behaviours than on relational dyads. Because married MSM may have a high risk of HIV infection, formulators of effective preventions should target this population, who may play a role in bridging HIV transmission.

Men who perceive their local HIV prevalence to be higher than the actual prevalence are less likely to engage in CAI [26], but this likelihood could increase if the partners have significant trust and are intimate with one another [27]. We further identified that stronger social ties between the sexual dyads were correlated with a higher risk of engaging in CAI. Interestingly, male sex workers’ perceptions of their sexual partners’ HIV status are more likely to be accurate than other people’s perceptions [28]. Thus, existing interventions targeting special populations, especially male sex workers, are effective. We found that non-occasional sexual activities were associated with CAI; a suitable explanation may be that the perceived HIV risk was not associated with condom use among regular partners who do not know their HIV statuses, even though these behaviours could be considered risky [29]. This result is consistent with previous studies that found that regular MSM partners were at a high risk of HIV transmission [26] [30]. Therefore, it may be beneficial to inform the public about the HIV epidemic, including the HIV infection rate each year among different subpopulations.

Although several previous studies have been of high quality, the findings have been inconsistent and did not show clear evidence of a relationship between Internet sex-seeking and sexual risk [31]. We found that MSM who sought sex on the Internet were less likely to practice CAI than MSM who did not. As Internet sites and smartphone apps continue to become popular among MSM, Internet-based intervention activities could be a good way to target more MSM who are rarely reached by venue-based outreach [32]. That is, HIV health education via the Internet could be effective. More acceptable and innovative prevention programs should be developed using online technologies.

Our research has several important limitations. First, although we used a sampling methodology designed to identify a minimally biased sample of VCT-attending MSM in our survey, our respondents are not representative of all MSM in Guangzhou City or elsewhere. Second, we did not measure other sexual network characteristics, such as sexual network density, as independent variables incorporated into the regression model. The original research design included the sexual network density as an index in our questionnaire. Most participants did not answer the relevant questions. We should strengthen the surveyor’s skills in future studies. Thirdly, the sample size of older men (≥41) was smaller, therefore, the lack of statistical significance for comparisons could just be due to the lack of power. In addition, PrEP use was not legally available in Guangzhou during our investigation, therefore, we did not collect any information on PrEP use.

## Conclusion

Our study indicates that HIV-related risky behaviours, especially CAI, are associated with the strong ties of sex dyads and different mixing patterns across age, educational categories and marital statuses. These key determinants should be considered in developing intervention strategies and programs for AIDS prevention and control.

## Additional files

Additional file 1: Questionnaire for MSM in Guangzhou. Questionnaire for MSM in Guangzhou. (PDF 131 kb)

Additional file 2: The characteristics of communication between respondents and their sexual partners. The count and proportion of each communication activity. (PDF 45 kb)

## Data Availability

All data generated or analyzed during this study are included in this published article and its supplementary information files.

## Abbreviations

AIDS: Acquired Immune Deficiency Syndrome
AOR: Adjusted Odds Ratio
CAI: Condomless Anal Intercourse
CDC: Center for Disease Control and Prevention
CI: Confidence Interval
GEE: Generalized Estimating Eqs.
HIV: Human Immunodeficiency Virus
MSM: Men who have Sex with Men
NGOs: non-governmental organizations
SNA: Social Network Analysis
STS: Social tie strength
UNAIDS: The Joint United Nations Programme on HIV/AIDS
VCT: HIV Voluntary Counseling & Testing
WT: Weak Tie
