# Supplementary material for: The characteristics of mixing patterns of sexual dyads and factors correlated with condomless anal intercourse among men who have sex with men in Guangzhou, China
Source: BMC Public Health. 2019 Jun 10;19:722. doi: 10.1186/s12889-019-7082-9 (PMC6558892; doi:10.1186/s12889-019-7082-9)
Supplement: Supplementary file 1 — Questionnaire for MSM in Guangzhou. Questionnaire for MSM in Guangzhou. (PDF 131 kb) [file 12889_2019_7082_MOESM1_ESM.pdf]

Questionnaire for MSM in Guangzhou

ID：_____

Please type "√" on the option or fill in the blank

Q1.What is your birthday？ _____year_____month

Q2.What is your education degree?

① Primary school and below
② Middle school
③ High school
④ Undergraduate
⑤ Master’s and above

Q3.What is your current marital status?

① Married
② Unmarried
③ Divorced
④ Other

Q5. What is your income in last year?

① <20000
② 20000-
③ 60001-
④ 120001-
⑤ >240000

Let's talk about your male sexual partners. Please list their initials or nicknames in order, including the following:

（Please fill in the option number of the corresponding answer in the option box, please specify the "Others" option.）

List(initials or nicknames)	Q7. the duration of the social interaction with this guy	Q8. the frequency of the social interaction with this guy	Q9. the affective interaction with this guy	Q10. how many times they had had sex with this partner in last 12 months (fellatiob and anal sex)?	Q11. whether you have had condomless insertive anal intercourse without using HIV-PrEP (Pre-Exposure Prophylaxis) with this guy in the past 12 months?	Q12. Do you meet with your sexual partners via dating software or websites?	Q13. where did you meet each other when you were first dating?	Q14.how old is your friend?	Q15.what is the education degree of this guy?	Q17. What is the current marital status of this guy?	Q18. What is the income of this guy in last year
	1.just met<br>2.0.5-1years<br>3.1-3years<br>4.3-10years<br>5.above10years	1.just once<br>2.at least once each year<br>3.at least once each month<br>4.at least once each week<br>5.almost everyday	1.seldom<br>2.at least once each year<br>3.at least once each month<br>4.at least once each week<br>5.almost everyday	1.just once<br>2.less than once each month<br>3.at least once each month<br>4.at least once each week<br>5.almost every day	1.never used a condom<br>2.seldom used condoms<br>3.intermittent used condoms<br>4.always used condoms	0.no<br>1.yes	1. wine bar or KTV<br>2. chess room or tea room<br>3. public bathhouse or clubhouse<br>4. public park<br>5. private residence or hotel<br>6. shopping mall or a public recreational venue	1.<17<br>2.18-25<br>3.26-30<br>4.31-40<br>5.41-50 岁<br>6.>51	1.Primary school<br>2.Middle school<br>3.High school<br>4.Undergraduate school<br>5.Master’s or above<br>6.Unknown	1.Married<br>2.Unmarried<br>3.Divorced<br>4.Other	1.<20000<br>2.20000-<br>3.60001-<br>4.120001-<br>5.>240000<br>6.Unknown
1											
2											
3											
4											
5											
6											
7											
8											
9											
10											
11											
12											
13											
14											
15											

Note: The content included in this questionnaire is a part of our whole study. We just post the content which is related to this paper.
