# Supplementary material for: The characteristics of mixing patterns of sexual dyads and factors correlated with condomless anal intercourse among men who have sex with men in Guangzhou, China
Source: BMC Public Health. 2019 Jun 10;19:722. doi: 10.1186/s12889-019-7082-9 (PMC6558892; doi:10.1186/s12889-019-7082-9)
Supplement: Supplementary file 2 — The characteristics of communication between respondents and their sexual partners. The count and proportion of each communication activity. (PDF 45 kb) [file 12889_2019_7082_MOESM2_ESM.pdf]

The characteristics of communication between respondents and their sexual partners

Variable	Label	Value	Frequency Count	Percent of Total Frequency
condom-fre	the frequencu of condom use	1	476	17.8478
		2	159	5.9618
		3	474	17.7727
		4	1558	58.4177
finteraction	frequency of the social interaction with the partner	3	682	25.5718
		4	535	20.06
		1	526	19.7225
		5	481	18.0352
		2	443	16.6104
inter	meet with your sexual partners via internet	0	607	10.8361
		1	2060	77.2403
intimacy	the affective interaction with the partner	1	919	34.4582
		3	557	20.8849
		4	417	15.6355
		2	416	15.5981
		5	358	13.4233
pm	place of meeting sexual partner	5	856	32.096
		7	738	27.6715
		6	492	18.4477
		4	194	7.2741
		1	143	5.3618
		8	99	3.712
		2	85	3.1871
		3	60	2.2497
dur	duration of the social interaction	2	1047	39.2576
		1	680	25.4968
		3	661	24.7844
		4	255	9.5613
		5	24	0.8999
sex-fre	frequency of sex	1	1189	44.5819
		2	647	24.2595
		3	458	17.1729
		4	341	12.7859
		5	32	1.1999
nominated	the number of the sexual partners of each respondant	3	707	26.5092
		4	694	26.0217
		5	547	20.5099
		6	201	7.5366
		7	115	4.312

	2	108	4.0495
	9	71	2.6622
	10	63	2.3622
	8	51	1.9123
	11	39	1.4623
	13	22	0.8249
	1	20	0.7499
	18	18	0.6749
	16	11	0.4124
